# Strigolactones are involved in phosphate- and nitrate-deficiency-induced root development and auxin transport in rice

**DOI:** 10.1093/jxb/eru029

**Published:** 2014-03-04

**Authors:** Huwei Sun, Jinyuan Tao, Shangjun Liu, Shuangjie Huang, Si Chen, Xiaonan Xie, Koichi Yoneyama, Yali Zhang, Guohua Xu

**Affiliations:** ^1^Key Laboratory of Plant Nutrition and Fertilization in Low-Middle Reaches of the Yangtze River, Ministry of Agriculture, Nanjing Agricultural University, Nanjing 210095, China; ^2^Weed Science Center, Utsunomiya University, 350 Mine-machi, Utsunomiya 321-8505, Japan

**Keywords:** Auxin, nitrate, phosphate, rice, root, strigolactone.

## Abstract

Strigolactones (SLs) or their derivatives have recently been defined as novel phytohormones that regulate root development. However, it remains unclear whether SLs mediate root growth in response to phosphorus (P) and nitrogen (N) deficiency. In this study, the responses of root development in rice (*Oryza sativa* L.) to different levels of phosphate and nitrate supply were investigated using wild type (WT) and mutants defective in SL synthesis (*d10* and *d27*) or insensitive to SL (*d3*). Reduced concentration of either phosphate or nitrate led to increased seminal root length and decreased lateral root density in WT. Limitation of either P or N stimulated SL production and enhanced expression of *D10*, *D17*, and *D27* and suppressed expression of *D3* and *D14* in WT roots. Mutation of *D10*, *D27*, or *D3* caused loss of sensitivity of root response to P and N deficiency. Application of the SL analogue GR24 restored seminal root length and lateral root density in WT and *d10* and *d27* mutants but not in the *d3* mutant, suggesting that SLs were induced by nutrient-limiting conditions and led to changes in rice root growth via *D3*. Moreover, P or N deficiency or GR24 application reduced the transport of radiolabelled indole-3-acetic acid and the activity of *DR5::GUS* auxin reporter in WT and *d10* and *d27* mutants. These findings highlight the role of SLs in regulating rice root development under phosphate and nitrate limitation. The mechanisms underlying this regulatory role involve *D3* and modulation of auxin transport from shoots to roots.

## Introduction

The ability of plants to sense the availability of soil nutrients and to respond accordingly is of fundamental importance for their adaptation to the environment. The plasticity of root development in response to nitrogen (N) or phosphorus (P) deficiency is vital, as N and P are two major nutrients required for plant growth and development (Forde and Lorenzo, 2001; López-Bucio *et al.*, 2003). Increased root-to-shoot ratio and root surface area induced by deficiency of N and P has been reported for several plant species (López-Bucio *et al.*, 2003; Chun *et al.*, 2005; Gruber *et al.*, 2013). Changes in root morphology under conditions of nutrient deficiency are complex and vary according to experimental conditions and plant species. Various plant growth studies have concentrated on the responses of root systems of different species under conditions of phosphate deficiency. A significant phosphate deficiency-induced response in *Arabidopsis thaliana* involves reduction of primary root growth and enhancement of lateral root (LR) density (Williamson *et al.*, 2001; Linkohr *et al.*, 2002; Chevalier *et al.*, 2003; López-Bucio *et al.*, 2003; Pérez-Torres *et al.*, 2008). In contrast to *Arabidopsis*, elongation of primary roots is a typical response to phosphate deprivation in other plant species (e.g. rice, *Oryza sativa* L.) (Yi *et al.*, 2005; Niu *et al.*, 2012). Little attention has been paid to root growth under conditions of N stress, possibly because of the inconsistent response of primary root length to nitrate deprivation depending on plant age and N concentration supplied in *Arabidopsis* (Zhang and Forde, 1998; Linkohr *et al.*, 2002; Gruber *et al.*, 2013). However, primary roots of rice and maize (*Zea mays*) typically elongate in response to N deprivation (Chun *et al.*, 2005; Tian *et al.*, 2008; Zhang *et al.*, 2012). Relative to phosphate deficiency, root morphology in rice under conditions of N limitation has not yet been characterized in detail.

Root formation is regulated by both environmental conditions and intrinsic factors (e.g. plant hormones). Auxins play a key role in establishing and developing patterns of root morphology and are regulated by varying N and P supply (Forde, 2002; Chiou and Lin, 2011; Niu *et al.*, 2012). Auxin signalling is associated with changes in root system architecture caused by phosphate deprivation; phosphate-deprived plants were shown to undergo primary root growth arrest and show formation of LR in the presence of exogenous auxin, while phosphate-supplied plants did not (López-Bucio *et al.*, 2003; Chiou and Lin, 2011; Shen *et al.*, 2013). In a previous study, inhibition of root growth in maize by high nitrate concentration was closely related to a reduction of auxin level in roots, and exogenous 1-naphthaleneacetic acid (NAA) and indole-3-acetic acid (IAA) restored primary root growth in the presence of high concentrations of nitrate (Tian *et al.*, 2008). Few studies have evaluated the role of auxin in regulating root growth under low-N conditions.

In addition to auxin, strigolactones or their derivatives (SLs) play a pivotal role in modulation of root development (Kapulnik *et al.*, 2011a; Ruyter-Spira *et al.*, 2011; Rasmussen *et al.*, 2012). In tomato and *Arabidopsis*, LR densities are increased in SL-deficient and SL-insensitive mutants, suggesting that SLs affect LR formation (Koltai *et al.*, 2010; Kapulnik *et al.*, 2011b; Ruyter-Spira *et al.*, 2011). Application of the synthetic SL analogue GR24 resulted in decreased LR density via the signalling gene *MAX2*, which suppressed LR outgrowth. Ruyter-Spira *et al.* (2011) found that the net effect of SL on LRs is dependent on the auxin status of the plant. In contrast to findings in *Arabidopsis*, numbers of LRs on the seminal root did not differ among 14-d-old wild type (WT) and *d10* and *d14* rice mutants (Arite *et al.*, 2012). The detailed biological mechanisms by which SLs function as plant hormones to regulate root growth remain unclear.

Strigolactones are closely regulated by phosphate availability in the soil, as demonstrated in both legumes and nonlegumes (Yoneyama *et al.*, 2007, 2012). Strigolactones are thought to be involved in an adaptive response to phosphate deficiency because elevated SL levels in roots and root exudates under low-phosphate conditions may contribute to increased mycorrhizal colonization and nodulation, which enable plants to collect more nutrients (Yoneyama *et al.*, 2007; Xie *et al.*, 2010). Arite *et al.* (2012) reported that application of GR24 induced root elongation in rice, suggesting that positive regulation of rice root elongation by SLs might be another strategy by which rice plants adapt to phosphate deficiency. Further studies are needed to understand the mechanisms responsible for the effects of SLs on growth responses of rice roots under conditions of low phosphate availability. Similarly, the production of SLs by plants (primarily nonlegumes) is regulated by N availability regardless of different N forms (Yoneyama *et al.*, 2007). Given elevated SL levels in rice and greater root elongation under conditions of N deficiency (Jamil *et al.*, 2011a; Zhang *et al.*, 2012), it is of interest to explore whether SLs contribute to the architecture of the rice root system under conditions of N starvation.

In this study, the role of SLs in regulating rice root growth in response to P- and N-limiting conditions was examined in SL-deficient and SL-insensitive mutants. Rice is an ideal model for SLs study because it has a range of well-characterized SL-synthesis mutants (*d10*, *d17*, and *d27*) and SL-response mutants (*d3*, *d14*, and *d53*) (Supplementary Fig. S1 available at *JXB* online; Xie *et al.*, 2010; Waters *et al.*, 2012; Jiang *et al.*, 2013; Zhou *et al.*, 2013). The current study found that root growth was related to elevated SL levels in roots and that exogenous GR24 restored seminal root length and reduced LR density under conditions of P and N deficiency in WT plants and SL-synthesis mutants, but not in the SL-signalling mutant *d3*. In addition, SL-deficient rice mutants carrying the auxin reporter construct *DR5::GUS*, and polar transport of radiolabelled [^3^H] IAA, revealed that SLs affect root growth in rice by decreasing auxin transport from shoots to roots.

## Materials and methods

### Plant growth

Wild-type rice (*O. sativa* L.) used in this study was the Shiokari ecotype. The SL-deficient mutants (*d10* and *d27*, respectively) and the SL-signalling mutant (*d3*) were kindly provided by Shinjiro Yamaguchi of RIKEN Plant Science Center. Plants were grown in a greenhouse under natural light at day/night temperatures of 30/18°C. Seven-d-old seedlings of uniform size and vigour were transplanted into holes in a lid placed over the top of pots (four holes per lid and three seedlings per hole). Nutrient solutions varying from one-quarter to half strength were applied for 1 week and then full-strength nutrient solution was applied for an additional 2 weeks. Pots receiving phosphate treatments were filled with solutions lacking P or containing varying phosphate concentrations (1, 2, 10, 100, and 300 μM). Pots receiving nitrate treatments were filled with solutions lacking N or containing 0.01, 0.02, 0.1, 1, or 2.5mM nitrate. The full chemical composition of the International Rice Research Institute (IRRI) nutrient solution was 1.25mM NH_4_NO_3_, 0.3mM KH_2_PO_4_, 0.35mM K_2_SO_4_, 1.0mM CaCl_2_, 1.0mM MgSO_4_.7H_2_O, 0.5mM Na_2_SiO_3_, 20.0 μM Fe-EDTA, 9.0 μM MnCl_2_, 0.39 μM (NH_4_)_6_Mo_7_O_24_, 20.0 μM H_3_BO_3_, 0.77 μM ZnSO_4_, and 0.32 μM CuSO_4_ (pH 5.5). Each treatment consisted of four replicates arranged in a completely randomized design to avoid edge effects. The nutrient solution was replaced with fresh solution daily.

Phosphate was supplied in the nutrient medium as KH_2_PO_4_. To exclude potential effects of potassium (K^+^) on the treatments, the low-phosphate treatment solutions were supplemented with K^+^ to the same levels as those under sufficient phosphate conditions (300 μM) using K_2_SO_4_. Nitrate was supplied in the nutrient medium as Ca(NO_3_)_2_. Similarly, concentrations of Ca^2+^ in the low-nitrate treatment solutions were supplemented to the same levels as those under sufficient nitrate conditions (2.5mM) using CaCl_2_. In the experiments, the N forms, NH_4_^+^/NO_3_^–^ in phosphate treatments and NO_3_^–^ in NO_3_^–^ treatments, were different from each other. As shown in Supplementary Fig. S2 available at *JXB* online, under two N concentrations (0.02 and 2.5mM), no difference was recorded in seminal root length and LR density between NH_4_^+^/NO_3_^–^ and NO_3_^–^ treatments.

Hormonal treatments, including GR24 (dissolved in acetone) and NAA (dissolved in 1M NaOH), were applied to the plant-growth medium since P and N treatments starting. Seven-d-old rice seedlings were grown in solutions containing varying levels of phosphate and nitrate with 2.5 μM GR24 or 10nM NAA. GR24 treatments were applied in the same IRRI nutrient solution in 0.4% Phytogel medium. Rice seeds were germinated in trays for 3 d and then transferred into plant culture tubes and grown for 2 weeks. The control treatment for GR24 consisted of addition of 0.1% acetone to the medium.

All plant tissues were sampled 4h after the start of the light period to assay root morphology, N, P and auxin concentrations. For determination of IAA, roots samples were weighed, snap-frozen in liquid N_2_, and stored in a –80°C freezer.

### Measurement of root system architecture

Seminal roots were significantly longer than adventitious roots under our experimental conditions. Our preliminary experiment showed that the responses of seminal roots to different treatments was similar to those of adventitious roots, and the number of adventitious root did not change significantly during experimental period. Therefore, seminal roots and LRs of seminal roots were chosen to study the effects of N and P on the rice root system. The length of seminal roots was measured with a ruler. Length and density of LRs were measured using the WinRhizo scanner-based image analysis system (Regent Instruments, Montreal, QC, Canada). The LR density was calculated as LR number divided by the length of the seminal roots.

### Measurement of strigolactones

After rice plants were grown in the treatments for 2 weeks, root exudates (approximately 500ml) were collected at 24-h intervals as described previously (Yoneyama *et al.*, 2012; Xie *et al.*, 2013). Root exudates adsorbed on charcoal were eluted with acetone. After evaporation of the acetone *in vacuo*, the residue was dissolved in 50ml water and extracted three times with 50ml ethyl acetate. The ethyl acetate extracts were combined, washed with 0.2M K_2_HPO_4_ (pH 8.3), dried over anhydrous MgSO_4_, and concentrated *in vacuo*. These crude extracts were stored in sealed glass vials at 4 °C until use.

The concentrations of SLs in the root exudation were determined by LC-MS/MS as described previously (Xie *et al.*, 2013). Data acquisition and analysis were performed with MassLynx version 4.1 (Waters, Milford, MA, USA). All experiments were repeated three times and the data are presented as mean±SE.

### qRT-PCR analysis

Total RNA was isolated from the roots of rice seedlings. RNA extraction, reverse transcription, and quantitative reverse-transcription PCR (qRT-PCR) were performed as described previously (Chen *et al.*, 2012). Primer sets for *D* and *PIN* are listed in Supplementary Tables S1 and S2 available at *JXB* online.

### Determination of IAA

The concentrations of IAA in the first leaf, junction, and roots were determined as described previously (Song *et al.*, 2013). Fresh weight of samples was determined, followed immediately by freezing in liquid N_2_. Sample measurement of free IAA by HPLC were carried out according to Song *et al.* (2013). A standard IAA sample was obtained from Sigma-Aldrich (St Louis, MO, USA).

To detect patterns of IAA distribution in rice plants, the *pDR5::GUS* construct was transformed into WT and *d10* and *d27* mutants using *Agrobacterium tumefaciens* EHA105; this construct was kindly provided by Professor Ping Wu’s group at Zhejiang University, Hangzhou, China. The samples used for IAA analysis were also used for histochemical GUS staining. Tissues were treated with ethanol prior to observation to remove chlorophyll pigmentation. The stained tissues were photographed using an Olympus SZX2-ILLK stereomicroscope with a colour CCD camera (Olympus).

### [^3^H]IAA-transport assay

Shoot-to-root auxin transport was assayed according to Song *et al.* (2013). [^3^H]IAA solution was applied to the cut surface after rice shoots were removed 2cm above the root–shoot junction. The four root segments – i.e. root tip (RT, 0.5cm), the zone where LR initiation and emergence occurred (LI, 0.5–4.0cm), the LR elongation zone in which LRs were visible but their length was not as long as that of mature roots (LE, 4.0–8.0cm), and the mature LR zone (ML, 8.0–12.0cm), were incubated in scintillation solution for 18h in the dark and then sampled and weighed. [^3^H]IAA radioactivity was detected using a multipurpose scintillation counter (LS6500, Beckman-Coulter, Fullerton, CA, USA).

### Data analysis

Experimental data were pooled for calculation of means and standard errors (SE) and analysed by one-way analysis of variance (ANOVA) followed by least significant difference (LSD) to determine the significance of differences between individual treatments. All statistical procedures were conducted with SPSS version 11.0 (SPSS, Chicago, IL, USA). In all analyses, *P*<0.05 was taken to indicate statistical significance.

## Results

### Low phosphate and nitrate concentrations enhanced seminal and lateral root elongation and inhibited lateral root density

**Fig. 1.**
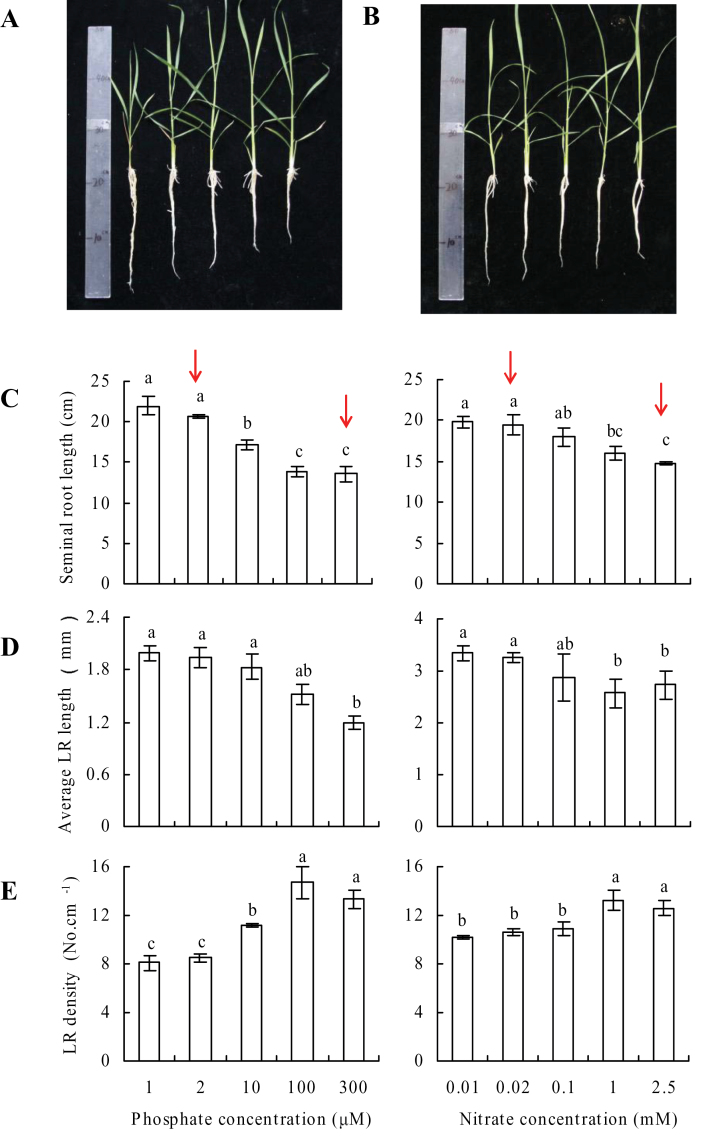
Effects of phosphate and nitrate availability on root morphology in wild-type rice seedlings. (A, B) Seedlings grown for 2 weeks in hydroponic media containing varying concentrations of phosphate (A) and nitrate (B). (C–E) Length of seminal root (C), lateral root (LR) length (D), and LR density (E) with containing varying concentrations of phosphate and nitrate. Red arrows indicate the four treatments for further study. Data are mean±SE. Different letters indicate significant differences (*P*<0.05, ANOVA) (this figure is available in colour at *JXB* online).

The length of seminal roots increased significantly in WT plants with decreasing phosphate concentration, from 300 to 1 μM (Fig. 1A). Seminal root length was 26% greater with 10 μM and approximately 55% greater in 1 and 2 μM phosphate compared to that under sufficient phosphate conditions (SP, 300 μM) (Fig. 1C–E). The average length of LRs in WT plants increased by approximately 64% with 1 or 2 μM phosphate, while LR density in WT decreased by 40% with low-P treatment relative to SP treatment.

The changes in WT root architecture with decreasing nitrate concentration were similar to those observed for phosphate (Fig. 1B). For example, seminal root length was 22% larger with 0.1mM nitrate and about 33% greater with 0.01 and 0.02mM nitrate compared to that under sufficient nitrate conditions (SN, 2.5mM; Fig. 1C–E). A 22% increase in LR length and 20% decrease in LR density were observed in WT plants grown with 0.01 or 0.02mM nitrate compared with SN treatment. Based on the simplicity and precision of its measurement, LR density was chosen as an indicator of LR growth for the subsequent experiments. Compared with 2.5mM nitrate (Supplementary Fig. S2 available at *JXB* online), N deficiency increased seminal root length and decreased LR density regardless of N forms.

Nitrogen and phosphorus concentrations were examined in WT plants grown in the presence of varying concentrations of these nutrients (Supplementary Fig. S3 available at *JXB* online). A significant decrease in P concentration was observed at phosphate concentrations <10 μM and a significant decrease in N concentration was observed at nitrate concentrations <0.1mM (Supplementary Fig. S3A available at *JXB* online). Compared to SP and SN, low phosphate (LP, 2 μM) decreased P concentration in rice plants to the same extent as the decrease in N concentration by low nitrate (LN, 0.02mM) (Supplementary Fig. S3B available at *JXB* online).

### Strigolactone evolution was enhanced in low-phosphate and low-nitrate media

**Fig. 2.**
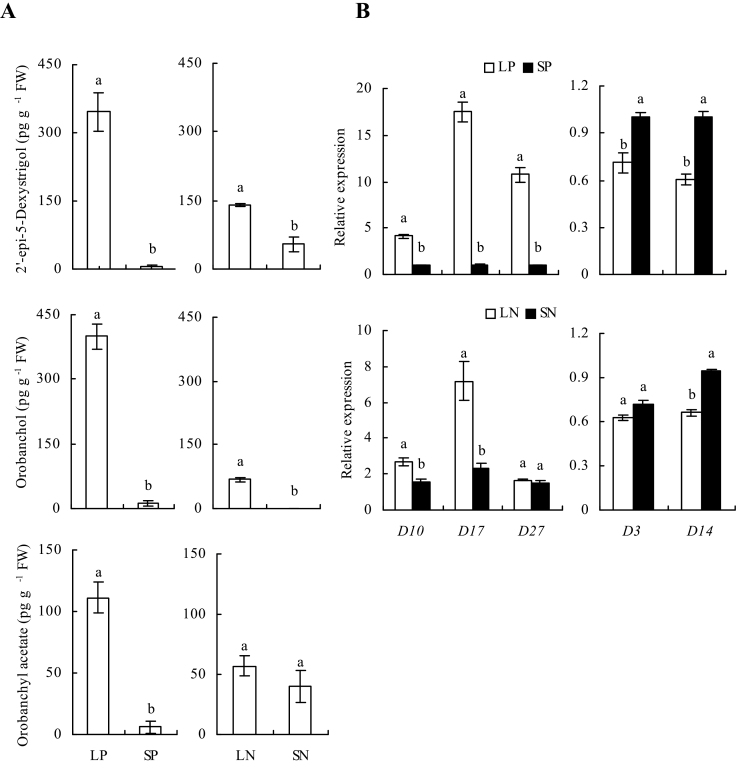
Levels of 2′-epi-5-deoxystrigol, orobanchol, and orobanchyl acetate exuded by wild-type rice plants (A) and qRT-PCR analysis of strigolactone-synthesis and -signalling genes (B) in wild-type rice plants. Seedlings were grown for 2 weeks in hydroponic media containing phosphate (LP, 2 μM; SP, 300 μM) and nitrate (LN, 0.02mM; SN, 2.5mM). Relative mRNA levels in B are normalized for individual gene relative to *OsACT*. Data are mean±SE. Different letters indicate significant differences (*P*<0.05, ANOVA).

To elucidate the role of SLs in the changes in root systems with low-phosphate and low-nitrate concentrations, SL exudates from WT roots were examined in WT plants. Compared with SP, the levels of 2′-epi-5-deoxystrigol, orobanchol, and orobanchyl acetate were increased by 63-, 33-, and 18-fold under LP conditions (Fig. 2A). Similarly, LN was associated with significantly enhanced levels of 2′-epi-5-deoxystrigol and orobanchyl acetate compared to SN. However, there was no difference in orobanchyl acetate content between LN and SN.

The expression patterns of SL-synthesis and SL-signalling genes were analysed in WT plants. The relative levels of *D10* and *D17* expression were significantly enhanced by LP and LN compared with SP and SN, while the relative expression of *D27* was enhanced only by LP (Fig. 2B). Interestingly, the expression levels of *D14* were lower under LP and LN than under SP and SN, respectively. It was only with LP compared with SP that *D3* expression was significantly lower. This effect may have been due to the higher SL levels in rice plants under LP and LN that downregulated the following signal gene as feedback, which is consistent with the previous report by Umehara *et al.* (2010).

### The *d10*, *d27*, and *d3* mutants were less sensitive than WT plants to low-phosphate and low-nitrate concentrations

**Fig. 3.**
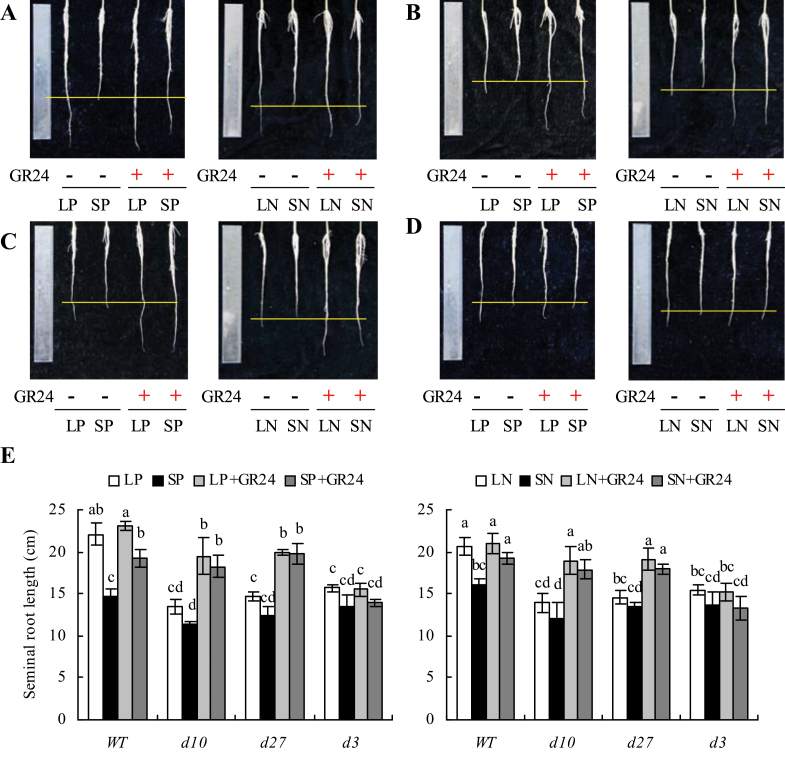
Effect of synthetic strigolactone analogue GR24 on seminal root length in wild type (WT, A), strigolactone-synthesis mutants *d10* (B) and *d27* (C), and the signalling mutant *d3* (D). Rice seedlings were grown for 2 weeks in agar media containing varying concentrations of phosphate (LP, 2 μM; SP, 300 μM) and nitrate (LN, 0.02mM; SN, 2.5mM) with or without 2.5 μM GR24. (E) Seminal root length. Data are mean±SE. Different letters indicate significant differences (*P*<0.05, ANOVA) (this figure is available in colour at *JXB* online).

**Fig. 4.**
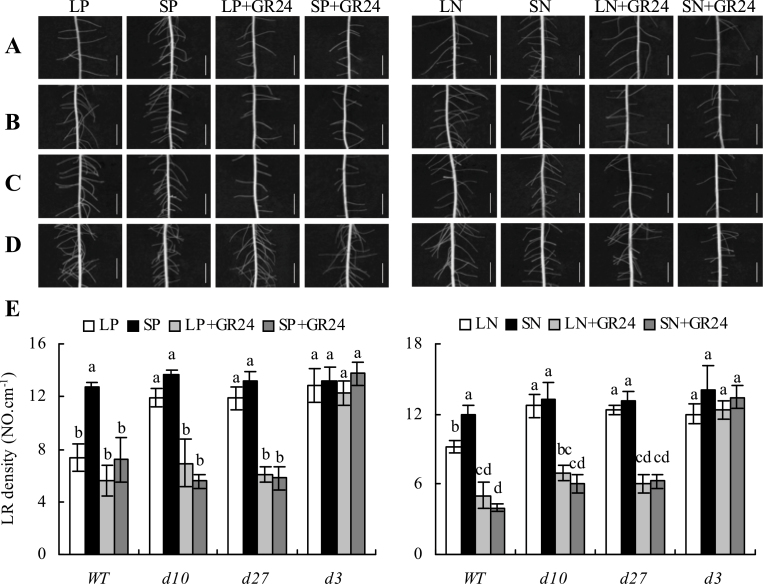
Effect of the-synthesis strigolactone analogue GR24 on lateral root (LR) density in wild type (WT, A), strigolactone-synthesis mutants *d10* (B) and *d27* (C), and the signalling mutant *d3* (D). Rice seedlings were grown for 2 weeks in the agar media under phosphate (LP, 2 μM; SP, 300 μM) and nitrate (LN, 0.02mM; SN, 2.5mM) concentrations with or without 2.5 μM GR24. Bars, 4mm. (E) LR density. Data are mean±SE. Different letters in the same gene indicate significant differences (*P*<0.05, ANOVA).

In contrast to WT plants, the root architectures of *d3*, *d10*, and *d27* were less responsive to LP and LN (Figs 3 and 4). For example, seminal root length was increased by 50% in WT plants and by approximately 18% in the three mutants when grown under LP (Fig. 3E). Seminal root length of WT plants grown in LN solution increased by 29%, while that of the mutants increased by approximately 14%. Similarly, LP and LN corresponded to reductions in LR density of 41 and 23%, respectively, in the WT (Fig. 4E). However, no differences in LR density were recorded in the three mutants under different nutrient conditions.

### Exogenous application of GR24 compensates for reduced response to low-phosphate and low-nitrate concentrations in mutants *d10* and *d27* but not in mutant *d3*

The effects of GR24 application to grown medium on root morphology were examined to assess whether the effects of LP and LN on rice root morphology were mediated by SLs. Rice plants grown under SP and SN in the presence of GR24 showed increased seminal root length and decreased LR density, starting from 1.25 μM GR24 (Supplementary Fig. S4 available at *JXB* online). Application of 2.5 μM GR24 had no effect on root morphology in WT plants under LP or LN conditions (Figs 3 and 4). However, application of GR24 in combination with SP and SN enhanced seminal root length and reduced LR density to the same extent as LP and LN. In *d10* and *d27* mutants, application of GR24 resulted in similar responses in seminal root length and LR density to those observed in WT plants under nutrient-deficient conditions. However, root morphology in the *d3* mutant did not respond to GR24 application, indicating the involvement of the *D3* gene family in the LP- and LN-regulating pathway.

### Auxin reduces changes in root systems induced by low-phosphate and low-nitrate concentrations

**Fig. 5.**
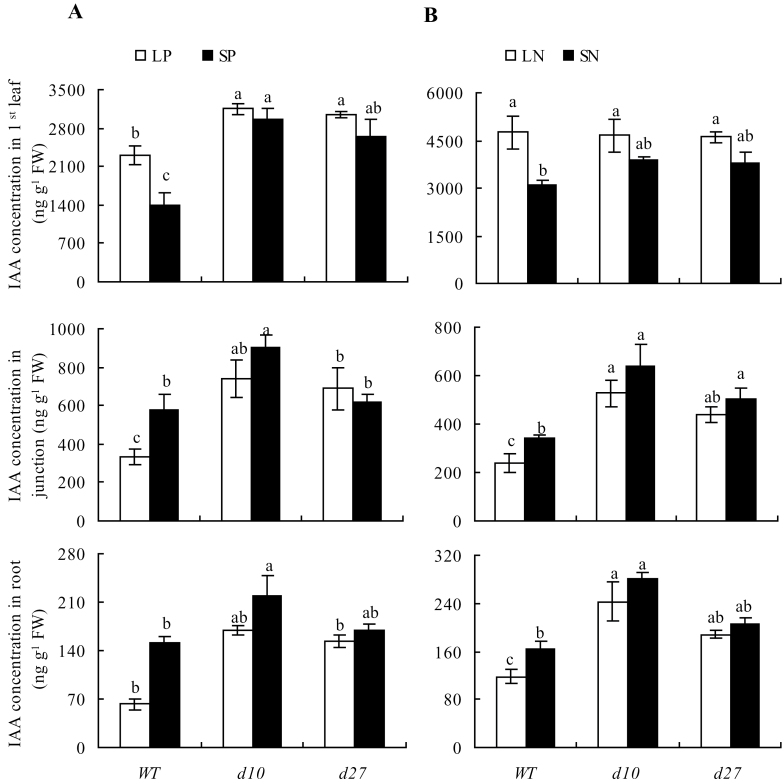
Auxin concentration in first leaf, junction, and root of wild type (WT) and strigolactone-synthesis mutants *d10* and *d27*. Rice seedlings were grown for 2 weeks in hydroponic media containing phosphate (A; LP, 2 μM; SP, 300 μM) and nitrate (B; LN, 0.02mM; SN, 2.5mM) concentrations. Data are mean±SE. Different letters indicate significant differences (*P*<0.05, ANOVA).

A 66% increase in IAA concentration was observed in the first leaf of WT seedlings under LP compared to SP; however, IAA concentrations in the junction and root were 43 and 58% lower in the LP treatment group, respectively (Fig. 5A). Similar trends in IAA concentration in WT seedlings were observed for the LN and SN treatments (Fig. 5B). These results indicated that LP and LN downregulated polar auxin transport from shoots to roots. In contrast, similar IAA concentrations were found in the tissues of *d10* and *d27* mutants under low and sufficient N and P, and higher IAA concentrations were found in mutant roots under LP and LN relative to the WT, which probably led to the shorter seminal roots observed in mutants.

**Fig. 6.**
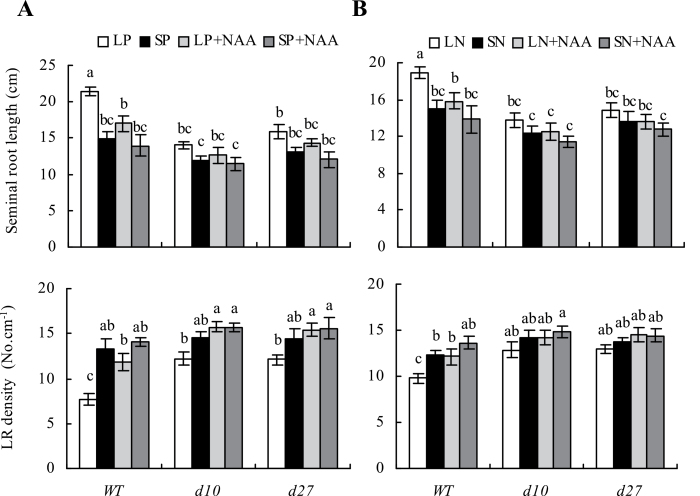
Effect of exogenous NAA application on seminal root length and lateral root (LR) density in wild type (WT) and strigolactone-synthesis mutants *d10* and *d27*. Seedlings were grown in hydroponic media containing phosphate (A: LP, 2 μM; SP, 300 μM) and nitrate (B: LN, 0.02mM; SN, 2.5mM) with or without 10nM NAA for 2 weeks. Data are mean±SE. Different letters indicate significant differences (*P*<0.05, ANOVA).

Wild-type plants grown under LN in the presence of 1–1000nM NAA showed a decrease in seminal root length with increasing NAA except at 1nM, at which a slight increase in seminal root length was observed (Supplementary Fig. S5 available at *JXB* online). Application of 10nM NAA counteracted the effects of phosphate and nitrate deficiency on root morphology in WT rice seedlings (Fig. 6). However, root morphology of *d10* and *d27* mutants grown under LP and LN was less sensitive to application of NAA (Supplementary Fig. S5 available at *JXB* online), probably due to higher endogenous IAA concentrations in the roots.

### Application of exogenous GR24 significantly reduces auxin transport under sufficient phosphate and nitrate concentrations

**Fig. 7.**
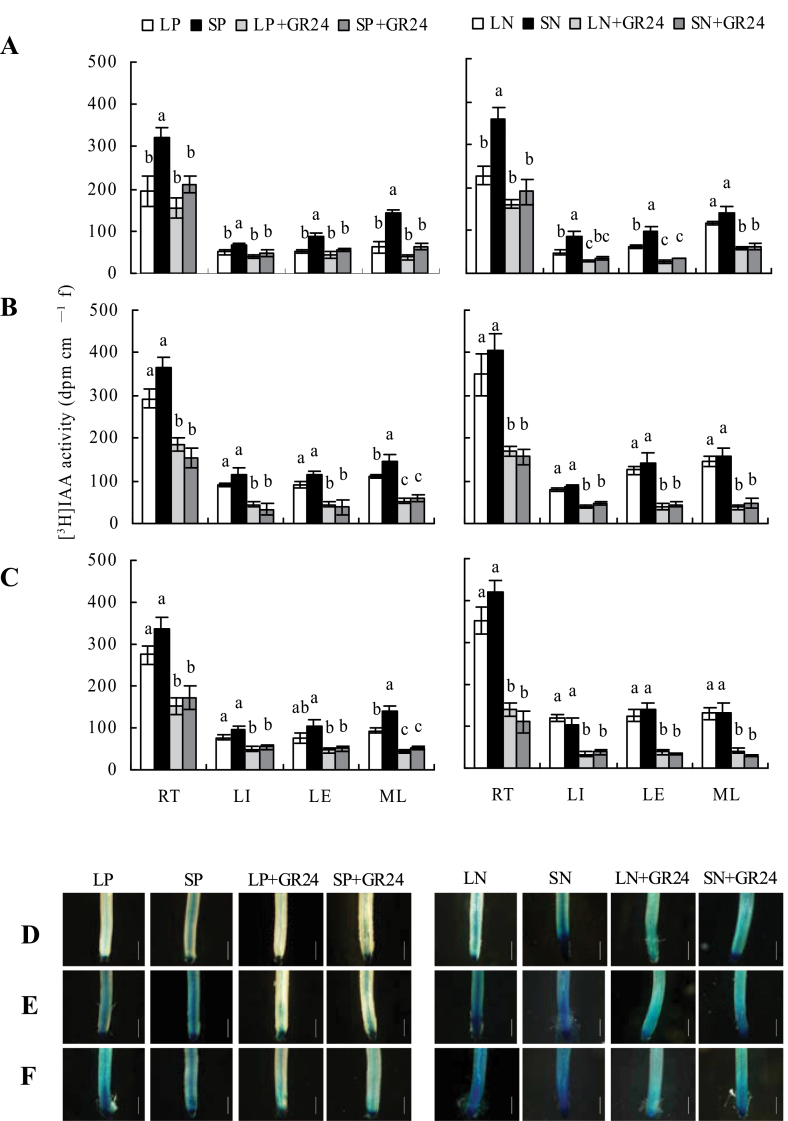
[^3^H]IAA transport (A–C) and histochemical localization of *DR5::GUS* activity (2h at 37 °C) in root tips (D–F). Seedlings were grown for 2 weeks in hydroponic media containing phosphate (LP, 2 μM; SP, 300 μM) and nitrate (LN, 0.02mM; SN, 2.5mM) with or without 2.5 μM GR24. (A, D) Wild type; (B, E) strigolactone-synthesis mutant d*10*; (C, F) strigolactone-synthesis mutant *d27*. RT, root tip; LI, lateral root initiation and emergence zone; LE, lateral root elongation zone; ML, mature lateral root zone. Bars, 1mm. Data are mean±SE. Different letters in the same root zone indicate significant differences (*P*<0.05, ANOVA) (this figure is available in colour at *JXB* online).

This study examined the effects of GR24 application on [^3^H]IAA transport (Fig. 7A–C). [^3^H]IAA transport from shoots to roots was significantly reduced under LP and LN in WT seedlings, which led to lower [^3^H]IAA activity in the four root zones. Application of GR24 to SP and SN treatments markedly reduced [^3^H]IAA transport in WT seedlings and in *d10* and *d27* mutants to the same extent as in WT seedlings under LP and LN.

**Fig. 8.**
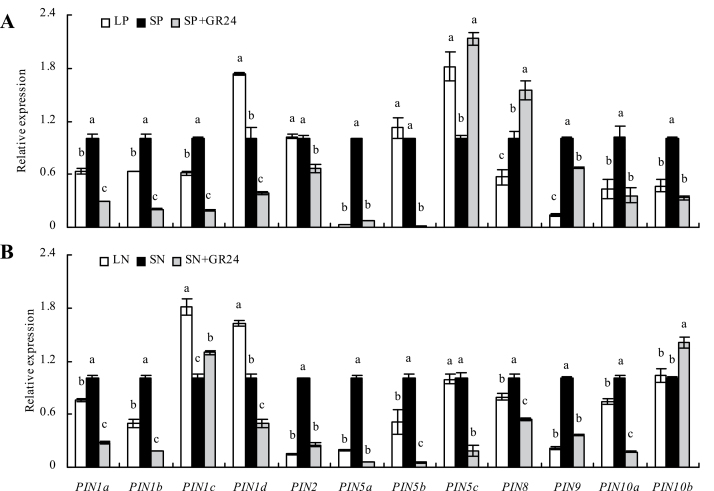
qRT-PCR analysis of *PIN* family genes in wild-type rice seedlings. Rice seedlings were grown for 2 weeks in hydroponic media containing phosphate (A: LP, 2 μM; SP, 300 μM) and nitrate (B: LN, 0.02mM; SN, 2.5mM). Relative mRNA levels were normalized for individual genes relative to *OsACT*. Data are mean±SE. Different letters in the same gene indicate significant differences (*P*<0.05, ANOVA).

The effects of GR24 on auxin status in rice were further investigated using transgenic plants expressing the *DR5::GUS* construct. In WT plants, *DR5::GUS* intensity was less widely distributed in root tips (Fig. 7D), 4 and 8cm from the root tip, junction, and stem (Supplementary Fig. S6 available at *JXB* online) under LP and LN conditions compared with SP and SN. No differences were recorded in mutants according to nutrient concentrations, and mutants showed higher *DR5::GUS* intensity than WT plants (Fig. 7D–F and Supplementary Fig. S6 available at *JXB* online), which was consistent with the results shown in Fig. 5. Application of GR24 under SP and SN significantly reduced IAA distribution in both WT and d10 and d27 mutants, and similar *DR5::GUS* intensity regulated by GR24 application was recorded in both WT and d10 and d27 mutants under respective nutrient-sufficient and -deficient conditions. These results further confirm that SLs induced by LP and LN downregulated polar auxin transport and consequently regulated root growth in rice.

qRT-PCR analysis showed that the expression levels of *PIN1a*-*b*, *PIN5a*, and *PIN8–10a* were significantly reduced under LP and LN compared to the respective nutrient-sufficient conditions (Fig. 8). Compared with respective nutrient-sufficient conditions, the levels of *PIN1c* and *PIN10b* expression were significantly reduced only in LP treatments, and *PIN2* and *PIN5b* expression were significantly reduced only under LN. However, expression of *PIN1d* and *PIN5c* increased significantly under LP and expression of *PIN1c-d* increased significantly under LN. Application of GR24 under SP and SN markedly downregulated the levels of most *PIN* family genes to levels similar to those under LP and LN, with the exception of *PIN5c* and *PIN8* under LP and *PIN1c* and *PIN10b* under LN. The expression of *PIN1a-b*, *PIN5a*, *PIN9*, and *PIN10a* was downregulated both by P and N deficiency and GR24 application.

## Discussion

Regulation of root growth in response to N and P deficiencies is essential for plants to optimize growth and productivity. Strigolactones have recently been defined as novel phytohormones that regulate root development (Kapulnik *et al.*, 2011a; Ruyter-Spira *et al.*, 2011; Mayzlish-Gati *et al.*, 2012; Rasmussen *et al.*, 2012). The present study provided evidence that the regulatory role played by SLs in rice root development under N- and P-limited conditions occurs via the *D3* gene component of SL signalling and by modulation of auxin transport from shoots to roots. These findings suggest that regulation of root growth by SLs may be a strategy for adapting to conditions of P and N deficiency in rice plants.

### Strigolactone exudation is enhanced by low-phosphate and low-nitrate concentrations

Unlike the universal promotion of SL production under conditions of P deficiency, increases in SLs under conditions of N deficiency rather than different N forms mostly occur in nonleguminous plants (Yoneyama *et al.*, 2007, 2012). In this study, the levels of the three main SL fractions in rice were markedly higher under LP than under SP. LN conditions corresponded to significant increases in the levels of 2′-epi-5-deoxystrigol and orobanchyl acetate and the expression levels of *D10* and *D17* compared to SN conditions. These findings are consistent with the exudation of SLs enhanced by N deficiency in a susceptible rice cultivar reported by Jamil *et al.* (2011a). Jamil *et al.* (2011a, b) found that SL production in response to nutrient deficiency differed markedly among rice cultivars.

Exudation of SL in relation to availability of N appears to be part of the plant nutrient acquisition strategy. Yoneyama *et al.* (2012) reported N deficiency decreased shoot P concentration in four upland plant species, which resulted in increased SL exudation. Interestingly, in this study, LN corresponded to decreased N concentrations in WT plants, whereas LP corresponded to decreases in both N and P concentrations; the decrease in N concentration was similar in plants grown under LP and LN (Supplementary Fig. S3B available at *JXB* online), consistent with the results of Cai *et al.* (2012). The decrease in N concentration under LP probably resulted from the approximately 70% lower P concentration in LP treatment, which may have affected ATP synthesis and the electrochemical potential of plant cell membranes, thus decreasing N uptake by rice plants.

### Rice root development is regulated by SLs via the *D3* component of SL signalling

Several lines of evidence suggest that the SL pathway is involved in root growth under conditions of low-phosphate and low-nitrate concentrations. First, under conditions of phosphate and nitrate deficiency, elevated SL exudation paralleled the changes in root architecture in WT rice plants. For example, higher levels of SL exudation in WT seedlings grown under LP than under LN conditions corresponded to longer seminal roots and lower LR density under LP (Figs 1 and 2).

Second, in tomato and *Arabidopsis*, LR density was increased in SL mutants, suggesting that SLs affect LR formation (Koltai *et al.*, 2010; Kapulnik *et al.*, 2011b; Ruyter-Spira *et al.*, 2011). In *Arabidopsis*, application of GR24 under SP conditions reduced LR formation by suppressing outgrowth of LRs, whereas application of GR24 under phosphate-limiting conditions induced LR formation (Kapulnik *et al.*, 2011b; Ruyter-Spira *et al.*, 2011). The density of LRs was affected by GR24 in WT seedlings and SL-synthesis mutants but not in the SL-signalling mutant, suggesting that the effect of SLs on LR density is mediated via the MAX2 F-box (Kapulnik *et al.*, 2011b; Koltai, 2011; Ruyter-Spira *et al.*, 2011). In contrast to findings in upland plants, LR density in rice did not differ among WT plants and *d* mutants in the present study (Fig. 4), consistent with the results of Arite *et al.* (2012). GR24 application under nutrient-sufficient conditions corresponded to reduced LR density, but the extent of the decrease did not change with increasing GR24 concentration (Supplementary Fig. S4 available at *JXB* online). Correspondingly, under nutrient-limiting conditions, application of GR24 did not lead to a further reduction in LR density, possibly because elevated SL levels under LN and LP may have been sufficient to affect LR initiation (Supplementary Fig. S4 available at *JXB* online). Moreover, application of GR24 caused recovery of the LR density phenotype induced by nutrient-limiting conditions in WT plants and *d10* and *d27* mutants but not in the *d3* mutant, further suggesting that elevated SL levels under low-nutrient conditions may lead to a *D3*-dependent reduction in LR density.

Third, in *Arabidopsis*, the effects of GR24 application on primary root length were dependent on growth conditions, such as sugar availability. (Koltai, 2011). Ruyter-Spira *et al.* (2011) GR24 application led to a *MAX2*-dependent increase in primary root length only when sucrose was omitted from the medium. However, in *Arabidopsis*, the involvement of SLs in this primary root response may not be associated with P status (Koltai, 2011). Interestingly, in the present study, application of GR24 led to increased seminal root length. Moreover, application of GR24 to WT plants and *d10* and *d27* mutants, but not the *d3* mutants under nutrient-sufficient conditions, led to complete recovery of the seminal root-length phenotype to that of WT plants under nutrient-limiting conditions, further confirming that elevation of SL levels under nutrient-limiting conditions can lead to a *D3*-dependent reduction in seminal root length.

### Development of rice roots is regulated by SLs via reduction of auxin transport from shoots to roots

There is accumulating evidence that auxins act as signalling intermediates in the responses of root architecture to low phosphate supply (Niu *et al.*, 2012). Polar auxin transport is essential for LR formation, but an auxin-transport-independent pathway is involved in changes in primary roots induced by phosphate stress in *Arabidopsis* (Williamson *et al.*, 2001; Linkohr *et al.*, 2002; López-Bucio *et al.*, 2003; Ticconi *et al.*, 2004; Jain *et al.*, 2009). Studies of *Arabidopsis* have provided insight into the crosstalk between auxin and the root growth response to P deficiency (Pérez-Torres *et al.*, 2008; Chiou and Lin, 2011). However, the mechanisms linking crosstalk between auxin and the response to N deficiency are poorly understood. In this study, IAA concentration was reduced under nutrient-limiting conditions in the junction and roots of WT plants, indicating that nutrient starvation prevented auxin transport from shoots to roots. In addition, application of NAA to WT plants under low-P and low-N conditions resulted in a phenotype that mimicked the root system architecture of WT plants under nutrient-sufficient conditions, indicating that auxin participated in regulation of root growth under nutrient-deficient conditions.

Strigolactones have been suggested to act as either modulators of auxin transport or as second messengers of auxin and to regulate shoot growth (Brewer *et al.*, 2009; Crawford *et al.*, 2010). In *Arabidopsis*, Ruyter-Spira *et al.* (2011) suggested that strigolactones are able to modulate local auxin levels and that the net result of strigolactone action is dependent on the auxin status of the plant. In present study, experiments with [^3^H]IAA transport and *DR5::GUS* activity further confirmed that application of GR24 markedly reduced auxin transport to levels equivalent to those under N- and P-deficient conditions, which in turn reduced the densities of LR and increased seminal root length.

Polar auxin transport is mediated primarily by *PIN* genes. Application of GR24 decreased the *PIN1*, *PIN3*, and *PIN7*-GFP gene expression levels in the primary root tip of *Arabidopsis*; however, *PIN* levels were unaffected when similar levels of GR24 were applied in the presence of exogenous auxin (Ruyter-Spira *et al.*, 2011). In the current study, which examined expression of 12 *PIN* family genes in rice roots, the expression of *PIN1a-b*, *PIN5a*, *PIN9*, and *PIN10a* was downregulated both by P- and N-deficiency and by GR24 application, indicating that they involved auxin transport from shoots to roots downregulated by SLs under nutrient deficiency.

In conclusion, elevated exudation of strigolactones was observed in WT plants under phosphate- and nitrate-limiting conditions relative to those under conditions of sufficient respective nutrient availability. Strigolactones regulated the development of rice roots via the *D3* component of SL signalling and by modulating transport of auxin from shoots to roots.

## Supplementary material

Supplementary data are available at *JXB* online.

Supplementary Table S1. Primers for qRT-PCR of *D* genes.

Supplementary Table S2. Primers for qRT-PCR of *PIN* genes.

Supplementary Fig S1. Summary of SL biosynthetic and signalling pathways.

Supplementary Fig S2. Root morphology in response to different N forms.

Supplementary Fig S3. Effect of phosphate and nitrate availability on phosphorus and nitrogen concentrations in wild-type plants.

Supplementary Fig S4. Root architecture in response to GR24 application.

Supplementary Fig S5. Root architecture in response to differing NAA supplies.

Supplementary Fig S6. *DR5::GUS* activity in stem, junction, and root.

Supplementary Data
