# Supplementary material for: Strigolactones are involved in phosphate- and nitrate-deficiency-induced root development and auxin transport in rice
Source: J Exp Bot. 2014 Mar 4;65(22):6735–46. doi: 10.1093/jxb/eru029 (PMC4246174; doi:10.1093/jxb/eru029)
Supplement: Supplementary Data [file supp_65_22_6735__index.html]

Strigolactones are involved in phosphate- and nitrate-deficiency-induced root development and auxin transport in rice — Strigolactones are involved in phosphate- and nitrate-deficiency-induced root development and auxin transport in rice — Supplementary Data 

# Strigolactones are involved in phosphate- and nitrate-deficiency-induced root development and auxin transport in rice

## Supplementary Data

Data files

**Files in this Data Supplement:**

- Supplementary Data - Supplementary Data
