# Supplementary material for: Strigolactones are involved in phosphate- and nitrate-deficiency-induced root development and auxin transport in rice
Source: J Exp Bot. 2014 Mar 4;65(22):6735–46. doi: 10.1093/jxb/eru029 (PMC4246174; doi:10.1093/jxb/eru029)
Supplement: Supplementary Data [file supp_eru029_jexbot114587_file001.pdf]

Table S1 The primers for qRT-PCR of *OsD10*, *OsD17*, *OsD27*, *OsD3* and *OsD14* genes.

Gene	Primer sequence
Os*D3*	5'-CTTTCCTGCTGTCCTCTT-3'
	5'-GTAGCCTCACCTCACTCG-3'
Os*D10*	5'-AGATTGTGGCGAGCGTGGAG-3'
	5'-AGGAGCGGAGGTTGTGGAGG-3'
Os*D14*	5'-TTGAACGACAGCGACTACCACG-3'
	5'-GAAGAGGGTGCGGCTGAACT- 3'
Os*D17*	5'-CGAAGGGAAAGGAGTGGC-3'
	5'-CG AACGGGAAGTAC GGGAG-3'
Os*D27*	5'-CCCCACTTCAGCCCTTTC-3'
	5'- GTCCATAGAC CACCAACG-3'
Os*Actin*	5'-CAACACCCCTGCTATGTACG-3'
	5'-CATCACCAGAGTCCAACACAA-3'

Table S2 The primers for qRT-PCR of *OsPIN* family genes.

Gene	Primer sequence
OsPIN1a	5'-TCATCTGGTCGCTCGTCTGC-3'
	5'-CGAACGTCGCCACCTTGTTC-3'
OsPIN1b	5'-TGCACCCTAGCATTCTCAGCA-3'
	5'-CCCTCCTCCCAAATTCTACTT-3'
OsPIN1c	5'-CCGTCAGGTTCCTCGTGGGT-3'
	5'-TCACGGCTGTGCTCAGAATG- 3'
OsPIN1d	5'-GATTCCGACGTCGTCTCGCTCG-3'
	5'-GTCGGGTTCCGCGACGACTGCA-3'
OsPIN2	5'-CAACACCTACTCCAGCCTC-3'
	5'-TGGACCAGTCAAGAACCTC-3'
OsPIN5a	5'-GGGGCTGGTGCTAAAGTTCG-3'
	5'-TGAGGTAGGGCTGCCTGTATG-3'
OsPIN5b	5'-GGGCAGCAGGAGAGGGTGATAG-3'
	5'-GAATCGGCAGAGAGATCAATGT-3'
OsPIN5c	5'-CTTCACCGCCGACCAGTGCGAC-3'
	5'-GTGATGCACCACGAGAACCCGC-3'
OsPIN8	5'-GTTCCACTATATGTAGCTATGATAC-3'
	5'-CAGTCAAACTTCTCTGCACAGC-3'
OsPIN9	5'-GATACAAGATAGCGTCGTTCTC-3'
	5'-ATGATGTCTGCGTGGACCT-3'
OsPIN10a	5'-GTTGGATTGAGATAGGCTGAGGAG-3'
	5'-ATGGCGACGAAGCGGTTGAT-3'
OsPIN10b	5'-TCCGATGCAGGGTTAGGC-3'
	5'-AGGATGGTAGCGTGGAGGTT-3'

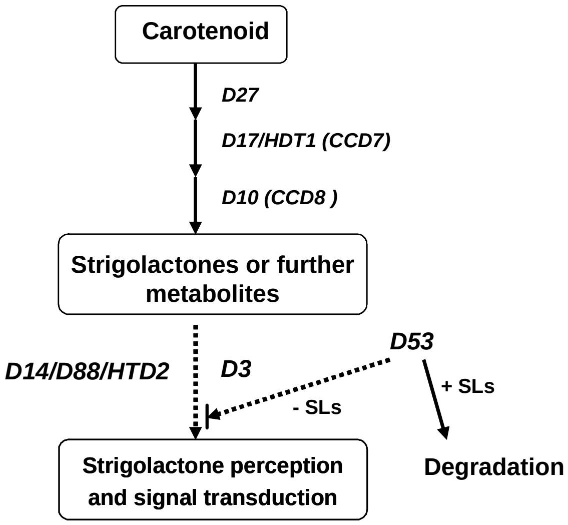

Fig. S1 Summary of strigolactone (SL) biosynthetic and signaling pathways in rice. The biosynthesis involves two carotenoid cleavage dioxygenases, CCD7 (*D17/HTD1*) and CCD8 (*D10*), and iron-containing protein (*D27*). SL signaling involves an F-box protein (*D3*), a member of α/β-hydrolase superfamily (D14/ D88/HTD2), and a protein sharing predicted features with the class I Clp ATPase proteins (*D53*). In the absence of SLs, D53 is stable and may recruit TPL/TPR proteins and repress downstream responses. In the presence of SLs, perception of SL leads to SCF^{D3}-mediated ubiquitination of D53 and its subsequent degradation by the proteasome system, which in turn releases the repression of downstream responses.

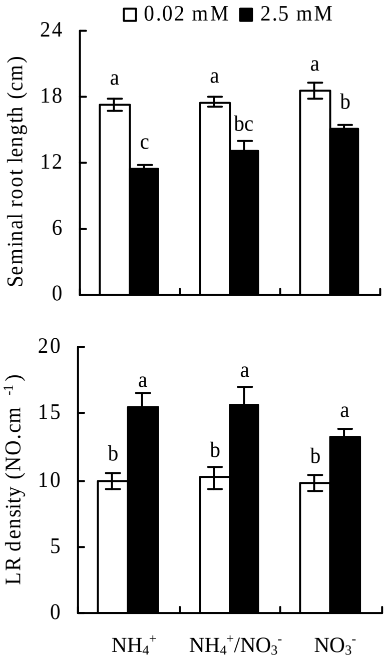

Fig. S2. Root morphology in wild-type rice plants under two nitrogen (N) concentrations varying the ratio of NH_4^+/NO_3^-. Seedlings were grown for two weeks in hydroponic media containing 0.02 and 2.5 mM N concentrations. Three NH_4^+/NO_3^- ratios were 100/0 (NH_4^+), 50/50 (NH_4^+/NO_3^-) and 0/100 (NO_3^-). Data are means ± SE and bars with different letters indicate significant difference at $P < 0.05$ tested with ANOVA.

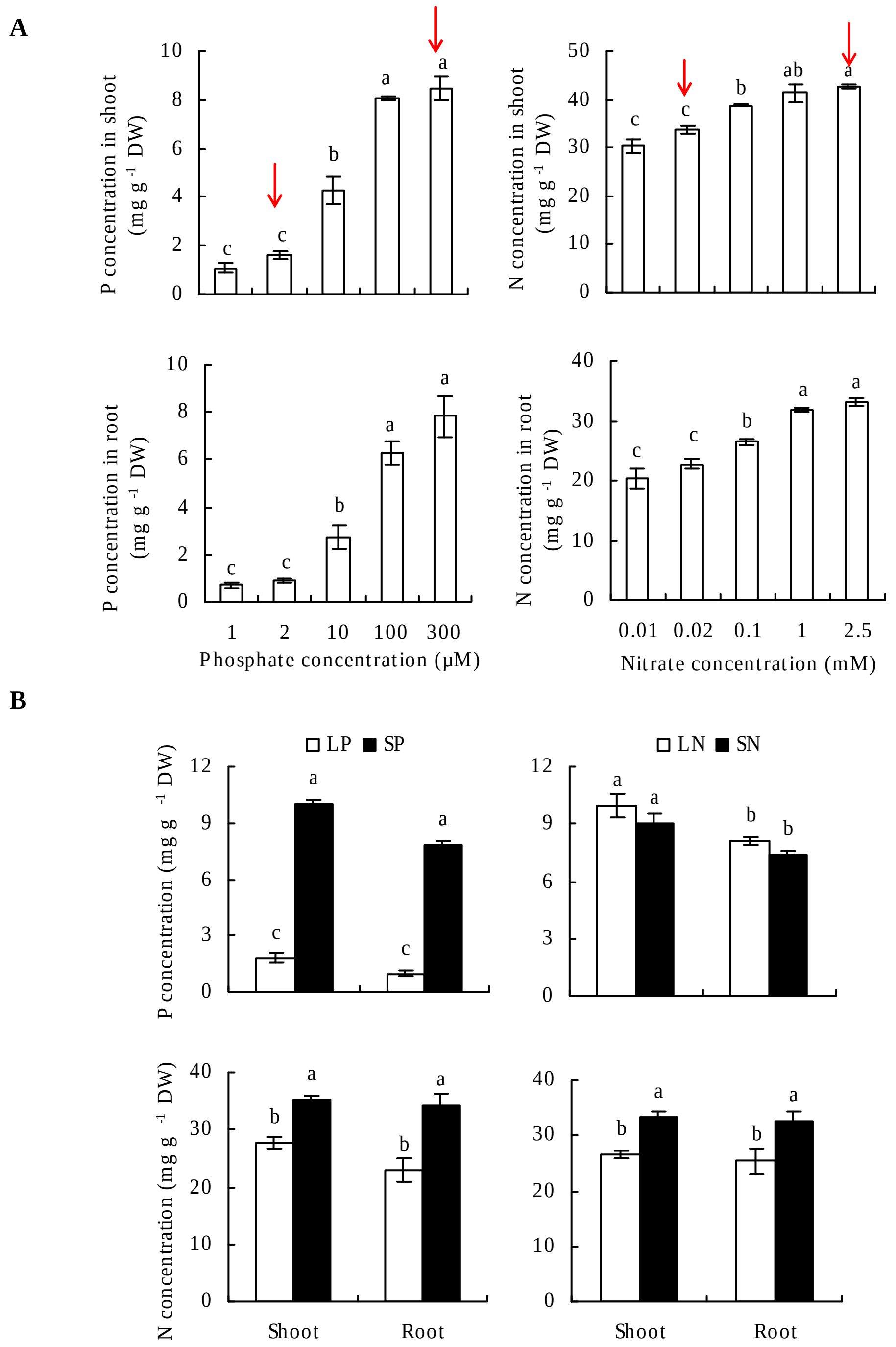

Fig. S3. Effect of phosphate and nitrate availability on phosphorus (P) and nitrogen (N) concentrations in the shoot and root of wild-type rice plants. Rice seedlings were grown for two weeks in hydroponic media containing varying concentrations of phosphate and nitrate(LP, 2 μM; SP, 300 μM) and nitrate (LN, 0.02 mM; SN, 2.5 mM). Red arrows showed the four treatments for further study. Data are means ± SE and bars with different letters indicate significant difference at $P < 0.05$ tested with ANOVA.

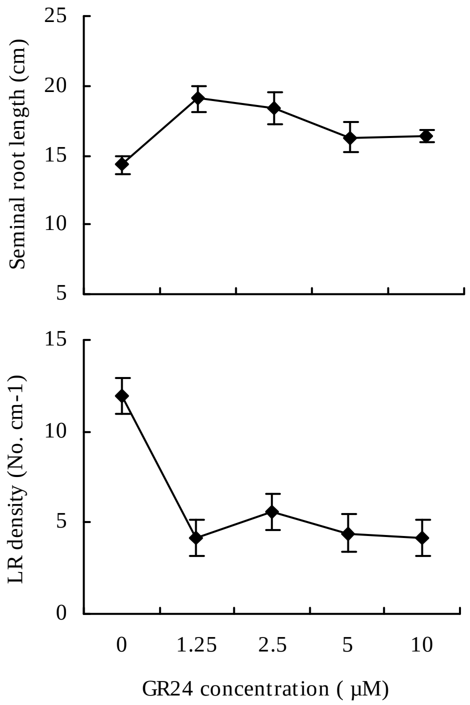

Fig. S4. Effect of synthetic strigolactone analog GR24 on root architecture in wild-type rice plants. Rice seedlings were grown for two weeks in agar media containing varying GR24 concentrations. Data are means ± SE and bars with different letters indicate significant difference at $P < 0.05$ tested with ANOVA.

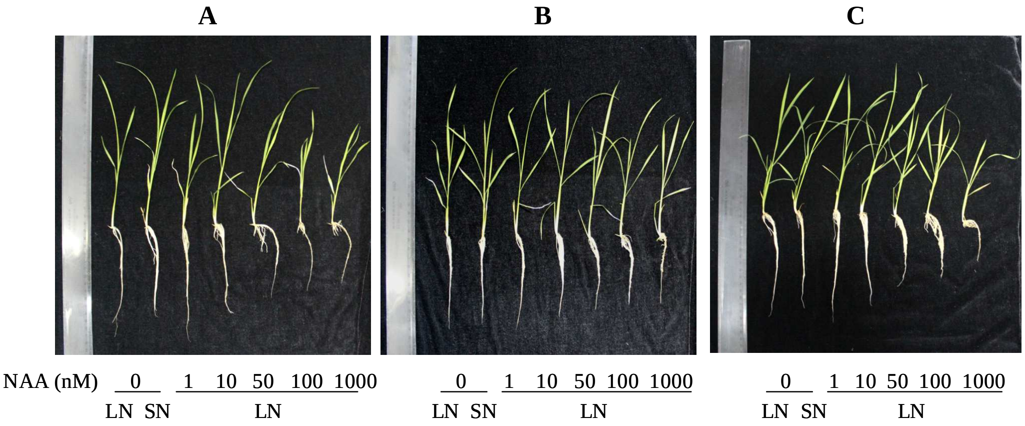

Fig. S5. Root architecture in wild-type (A), *d10* (B) and *d27* (C) rice subjected to differing NAA supplies. Rice seedlings were grown for two weeks in hydroponic media containing nitrate concentrations (0.02 mM, LN; 2.5 mM, SN) with or without NAA.

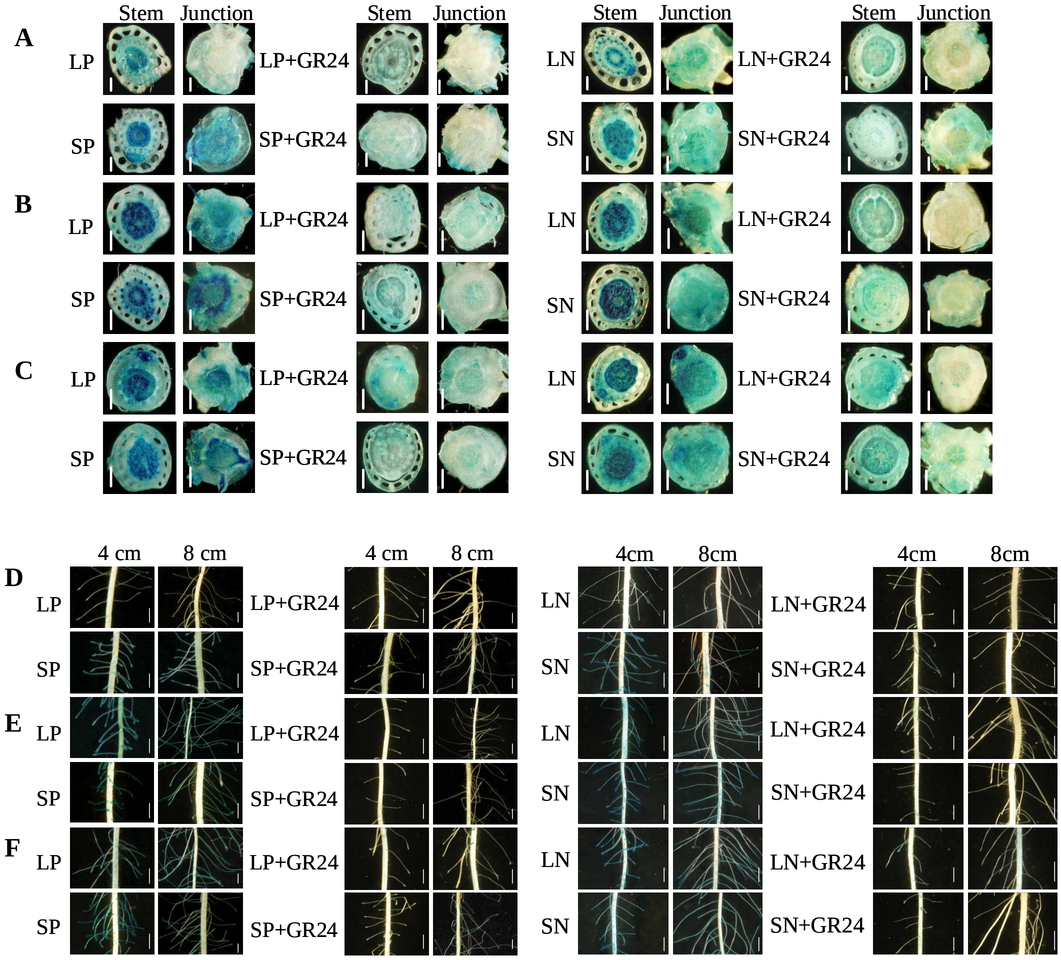

Fig. S6. Histochemical localization of *DR5::GUS* activity in stem and junction (A-C, Bar=0.5 mm) and in 4 cm and 8 cm from root tip (D-F, Bar=4 mm). (A, D) wild-type, (B, E) *d10* and (C, F) *d27* containing the *DR5::GUS* reporter construct. *DR5::GUS*, a specific reporter that contains seven repeats of a highly active synthetic auxin response element and can reflect the *in vivo* auxin level. Seedlings were grown for two weeks in hydroponic media containing phosphate (LP, 2 µM; SP, 300 µM) and nitrate (LN, 0.02 mM; SN, 2.5 mM) concentrations with or without 2.5 µM GR24. Plants were stained for GUS activity for 2 h under 37°C.
